# Preparation, Characterization and Intermediate-Temperature Electrochemical Properties of Er^3+^-Doped Barium Cerate–Sulphate Composite Electrolyte

**DOI:** 10.3390/ma12172752

**Published:** 2019-08-27

**Authors:** Fufang Wu, Ruifeng Du, Tianhui Hu, Hongbin Zhai, Hongtao Wang

**Affiliations:** 1 Anhui Provincial Key Laboratory for Degradation and Monitoring of Pollution of the Environment, School of Chemical and Material Engineering, Fuyang Normal University, Fuyang 236037, China; 2 Guangdong Provincial Key Lab of Nano-Micro Materials Research, School of Chemical Biology and Biotechnology, Peking University Shenzhen Graduate School, Shenzhen 518055, China

**Keywords:** composite, fuel cell, BaCeO_3_, conductivity, electrolyte

## Abstract

In this study, BaCe_0.9_Er_0.1_O_3−α_ was synthesized by a microemulsion method. Then, a BaCe_0.9_Er_0.1_O_3−α_–K_2_SO_4_–BaSO_4_ composite electrolyte was obtained by compounding it with a K_2_SO_4_–Li_2_SO_4_ solid solution. BaCe_0.9_Er_0.1_O_3−α_ and BaCe_0.9_Er_0.1_O_3−α_–K_2_SO_4_–BaSO_4_ were characterized by X-ray diffraction (XRD), scanning electron microscopy (SEM) and Raman spectrometry. AC impedance spectroscopy was measured in a nitrogen atmosphere at 400–700 °C. The logσ~log (*p*_O_2__) curves and fuel cell performances of BaCe_0.9_Er_0.1_O_3−α_ and BaCe_0.9_Er_0.1_O_3−α_–K_2_SO_4_–BaSO_4_ were tested at 700 °C. The maximum output power density of BaCe_0.9_Er_0.1_O_3−α_–K_2_SO_4_–BaSO_4_ was 115.9 mW·cm^−2^ at 700 °C, which is ten times higher than that of BaCe_0.9_Er_0.1_O_3−α_.

## 1. Introduction

With the rapid development of the economy, energy problems are imminent. As one of the new energy sources, fuel cells are of great significance. Solid oxide fuel cells (SOFCs) have the advantages of high conversion efficiency, small size, no noise, reduced pollution, and so on [1,2,3,4,5,6,7,8]. However, higher operating temperatures often lead to serious performance degradation, longer start-up times and expensive interconnecting sealing materials, which are considered the main obstacles to SOFCs’ commercialization. Therefore, it is urgent to explore SOFCs operating at intermediate temperatures (400–700 °C) and at high performance at the same time. Compared with oxygen ion-conductive SOFCs, proton-conducting SOFCs can operate at lower temperatures. An exploration of electrolyte materials with high protonic conductivities at 400–700 °C is of vital importance [9,10,11,12,13,14]. 

It is known that BaCeO_3_-based ceramics have good protonic conductivities at high temperatures (700–1000 °C) [15,16,17,18,19,20,21,22,23,24]. Using an electrolyte film and a composite electrolyte are two main ways to apply BaCeO_3_-based ceramics to intermediate-temperature SOFCs [25,26,27,28,29,30,31,32,33]. Tong and O’Hayre fabricated five different types of H_2_/air fuel cells using BaCe_0.7_Zr_0.1_Y_0.1_Yb_0.1_O_3−δ_ (BCZYYb), BaCe_0.6_Zr_0.3_Y_0.1_O_3−δ_ (BCZY63) and BaZr_0.8_Y_0.2_O_3−δ_ (BZY20) as electrolytes [26]. Liu et al. reported that a 30 wt.% In^3+^-doped barium cerate–70 wt.% Gd_0.1_Ce_0.9_O_2−δ_ composite electrolyte had a high conductivity of 3.42 × 10^−2^ S·cm^−1^ in wet hydrogen at 700 °C [27]. The conductivities of barium cerate-ceria-type composite electrolytes are similar to those of BaCeO_3_ doped with low-valent metal cations [27,28,29]. Park et al. investigated BaZr_0.85_Y_0.15_O_3−δ_ (BZY)-carbonate composite electrolytes, which had good intermediate-temperature electrochemical properties [32]. The literature has mainly focused on reporting carbonate [30,31,32,33] and chloride [34,35,36] composite electrolytes. Only a small number of reports on cerium dioxide–sulfate composite electrolytes have been reported [37]. It is well known that the stability of carbonate is weaker than that of sulfate. Until now, no literature has reported on the barium cerate–sulphate composite electrolyte.

In this study, we synthesized BaCe_0.9_Er_0.1_O_3-α_by a microemulsion method. Then, a BaCe_0.9_Er_0.1_O_3−α_–K_2_SO_4_–BaSO_4_ composite electrolyte was obtained by compounding it with a K_2_SO_4_–Li_2_SO_4_ solid solution. The characterization and intermediate-temperature (400–700 °C) electrochemical properties of BaCe_0.9_Er_0.1_O_3−α_ and BaCe_0.9_Er_0.1_O_3−α_–K_2_SO_4_–BaSO_4_ were investigated. 

## 2. Experimental

BaCe_0.9_Er_0.1_O_3−α_ was prepared by a microemulsion method. Firstly, Er_2_O_3_ was completely dissolved with concentrated nitric acid. Sixty milliliters of water was added to make Ba(CH_3_COO)_2_ and (NH_4_)_2_Ce(NO_3_)_6_ dissolve evenly. A mixture of cyclohexane, ethanol and polyvinyl alcohol (PVA) was added to the solution and stirred until it was completely emulsified to form Microemulsion A. Then, (NH_4_)_2_CO_3_, NH_4_OH, cyclohexane, ethanol and PVA were mixed evenly to form Microemulsion B [38,39]. Microemulsion B was slowly added to Microemulsion A. In the process of dropping, the number of white precipitates increased, and a large number of bubbles emerged at the same time. The precipitation was filtered and dried under an infrared lamp to obtain the precursor powder. Finally, the precursor was calcined in a high-temperature furnace at 1250 and 1550 °C for 6 h to obtain BaCe_0.9_Er_0.1_O_3−α_. 

In this experiment, molten K_2_SO_4_–Li_2_SO_4_ (1:1 mole ratio) was prepared in a muffle oven at 750 °C for 2 h [40]. Our previous studies indicated that the stability of 70 wt.% SrCe_0.9_Yb_0.1_O_3−α_–30 wt.% (Na/K)Cl was lower, though its conductivities were higher than 80 wt.% SrCe_0.9_Yb_0.1_O_3−α_–20 wt.% (Na/K)Cl [41]. Therefore, the BaCe_0.9_Er_0.1_O_3−α_ powders were evenly mixed with molten K_2_SO_4_–Li_2_SO_4_ powders in a weight proportion of 80%:20%. After being sieved and pressed, the disks were put into the muffle furnace heated at 750 °C for 2 h to obtain BaCe_0.9_Er_0.1_O_3−α_–K_2_SO_4_–BaSO_4_. 

BaCe_0.9_Er_0.1_O_3−α_ and BaCe_0.9_Er_0.1_O_3−α_–K_2_SO_4_–BaSO_4_ were characterized by an X-ray diffractometer (XRD, X’pert Pro MPD, Holland’s company, Amsterdam, Netherlands), a confocal-micro Raman spectrometer (invia, Renishaw, Gloucestershire, United Kingdom), and a scanning electron microscope (SEM, S-4700, Hitachi, Tokyo, Japan). The Ba, Ce, Er, O, K and S elements in BaCe_0.9_Er_0.1_O_3−α_–K_2_SO_4_–BaSO_4_ were measured by the energy-dispersive X-ray spectroscopy.

For intermediate-temperature electrochemical properties, BaCe_0.9_Er_0.1_O_3−α_ and BaCe_0.9_Er_0.1_O_3−α_–K_2_SO_4_–BaSO_4_ were polished to a thickness of 1.0 mm. Circles 8 mm in diameter were drawn in the center of both sides of the discs with a pencil, and a 20%Pd–80%Ag paste was coated on the circles (area: 0.5 cm^2^). AC impedance spectroscopy was measured in a nitrogen atmosphere at 400–700 °C. The frequency ranged from 1 to 10^5^ Hz, and the signal voltage was 0.05 V. The logσ~log (*p*_O_2__) curves of BaCe_0.9_Er_0.1_O_3−α_ and BaCe_0.9_Er_0.1_O_3−α_–K_2_SO_4_–BaSO_4_ were tested by adjusting different proportions of air, nitrogen, oxygen and hydrogen at room temperature (*p*_H2O_ = 2.3 × 10^3^ − 3.1 × 10^3^ Pa). The two sides of BaCe_0.9_Er_0.1_O_3−α_ and BaCe_0.9_Er_0.1_O_3−α_–K_2_SO_4_–BaSO_4_ were in hydrogen and oxygen atmospheres, respectively, which constituted the following fuel cells: H_2_, Pd–Ag | sample | Pd–Ag, O_2_. We then measured their *I*–*V*–*P* curves.

## 3. Results and Discussion

**Figure 1:**
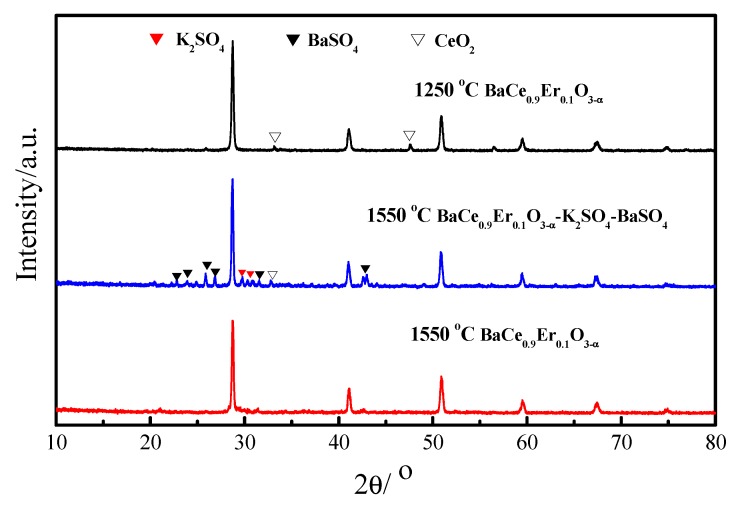
XRD patterns of BaCe_0.9_Er_0.1_Os_3-α_ (1250 and 1550 °C) and BaCe_0.9_Er_0.1_O_3−α_–K_2_SO_4_–BaSO_4_.

Figure 1 is the XRD spectra of BaCe_0.9_Er_0.1_O_3__−α_ (1250 and 1550 °C) and BaCe_0.9_Er_0.1_O_3−α_–K_2_SO_4_–BaSO_4_. The diffraction peaks of BaCe_0.9_Er_0.1_O_3−α_ (1250 and 1550 °C) correspond to the standard diagram of BaCeO_3_ (JCPDS 85-2155). Neither of the two samples detected Er_2_O_3_, which indicates that they had entered the lattice of perovskite phase. In BaCe_0.9_Er_0.1_O_3−α_ (1250 °C), in addition to the perovskite phase, there was a very small amount of the CeO_2_ phase, indicating the initial calcination temperature should be raised to 1300 or 1350 °C [22]. The weak alkali salt Li_2_SO_4_ reacted with the strong base BaO to form BaSO_4_ when BaCe_0.9_Er_0.1_O_3−α_ powders were mixed with molten sulphate, as indicated by the equation: $\mathrm{BaO}+{\mathrm{Li}}_{2}{\mathrm{SO}}_{4}={\mathrm{BaSO}}_{4}+{\mathrm{Li}}_{2}O$. CeO_2_ may be separated from the perovskite structure when sulphate and BaCe_0.9_Er_0.1_O_3−α_ form a composite electrolyte. This is why CeO_2_ also appears in BaCe_0.9_Er_0.1_O_3−α_–K_2_SO_4_–BaSO_4_.

**Figure 2:**
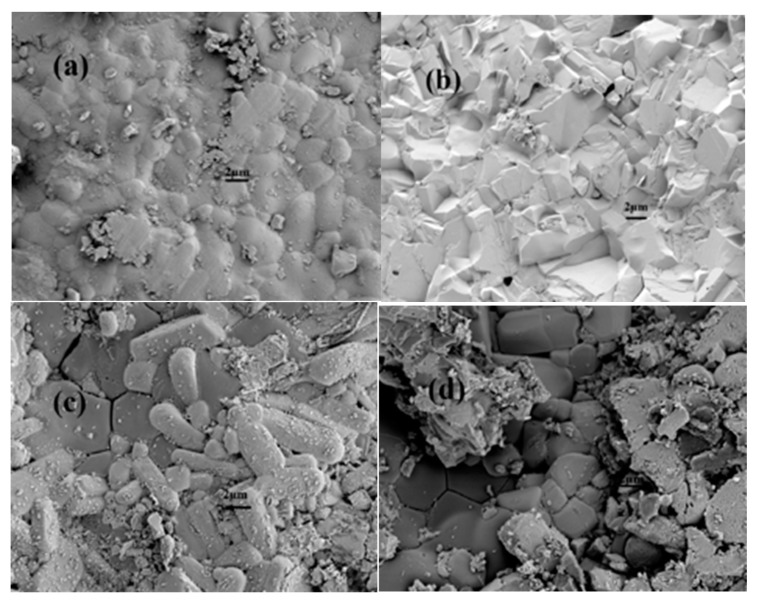
The external (**a**,**c**) and cross-sectional (**b**,**d**) SEM photos of BaCe_0.9_Er_0.1_O_3−α_ (1550 °C) and BaCe_0.9_Er_0.1_O_3−α_–K_2_SO_4_–BaSO_4_.

Figure 2a,b shows SEM photos of the external and cross-sectional surfaces of the BaCe_0.9_Er_0.1_O_3−α_ (1550 °C) ceramic prepared by the microemulsion method. It can be seen that BaCe_0.9_Er_0.1_O_3−α_ (1550 °C) had a compact structure, complete grain growth, clear grain boundaries, and very few holes. The density of the BaCe_0.9_Er_0.1_O_3−α_ (1550 °C) ceramic prepared by the microemulsion method was higher than that by the high-temperature solid-state method at the same sintering temperature. After adding sulphate, the boundaries between grains became not particularly distinct. There were different degrees of adhesion between grains [32,33]. This is due to the BaCe_0.9_Er_0.1_O_3−α_ grains being wrapped in molten sulfate.

**Figure 3:**
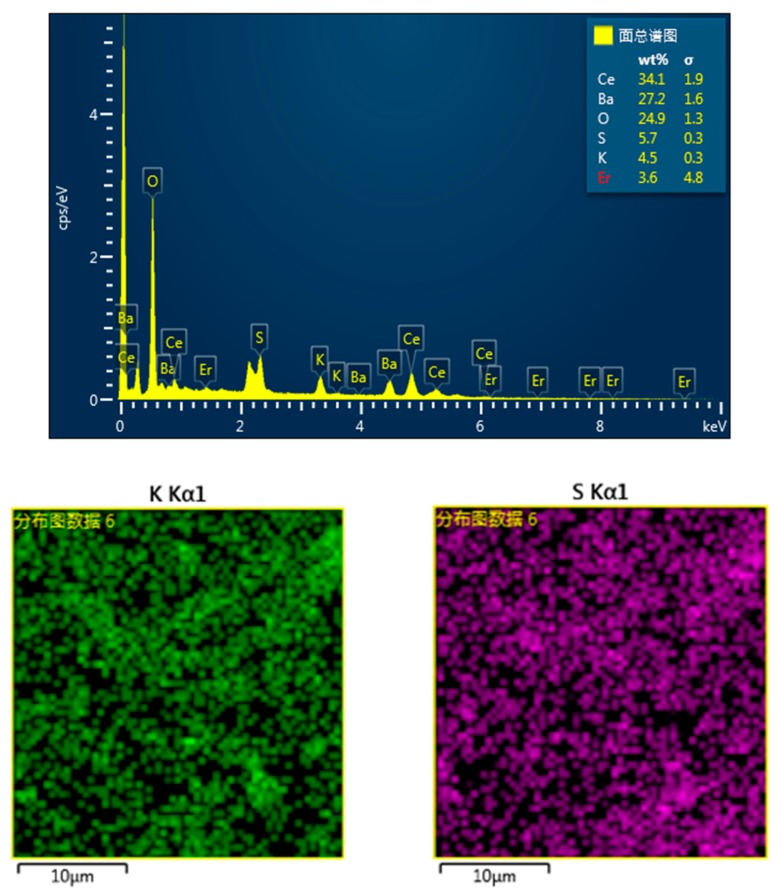
The energy-dispersive X-ray spectroscopy and elements mapping images in BaCe_0.9_Er_0.1_O_3−α_–K_2_SO_4_–BaSO_4_.

The energy-dispersive X-ray spectroscopy result of BaCe_0.9_Er_0.1_O_3−α_–K_2_SO_4_–BaSO_4_ is shown in Figure 3. The spectrum had major peaks assigned to the Ba, Ce, Er, O, K and S elements. The atomic ratios of Ba/Ce, Ba/Er and S/K are 0.87, 9.21 and 1.54. The low content of the Ba element may be due to the formation of BaSO_4_ by the reaction: $\mathrm{BaO}+{\mathrm{Li}}_{2}{\mathrm{SO}}_{4}={\mathrm{BaSO}}_{4}+{\mathrm{Li}}_{2}O$, resulting in segregation. The elements mapping images indicated that the spatial distribution of sulphate was uniform.

**Figure 4:**
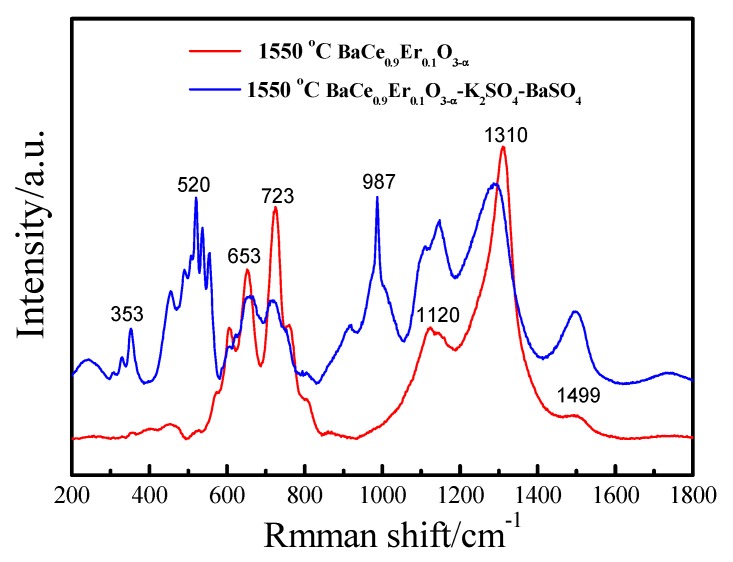
Raman spectra of BaCe_0.9_Er_0.1_O_3−α_ (1550 °C) and BaCe_0.9_Er_0.1_O_3−α_–K_2_SO_4_–BaSO_4_.

Figure 4 shows the Raman spectra of BaCe_0.9_Er_0.1_O_3−α_ (1550 °C) and BaCe_0.9_Er_0.1_O_3−α_–K_2_SO_4_–BaSO_4_. In BaCe_0.9_Er_0.1_O_3−α_ (1550 °C), Raman activity peaked around 653 and 723 cm^−1^, corresponding to the *O*_h_ vibrational mode and Ce–O vertical bending vibration in the *A*_1g_ mode, respectively. In BaCe_0.9_Er_0.1_O_3−α_–K_2_SO_4_–BaSO_4_, the Raman peaks near 353, 520, 987 and 1120 cm^−1^ were attributed to S–O bending, bending deformation, symmetrical stretching and antisymmetric telescopic vibration, respectively [38,42,43,44].

**Figure 5:**
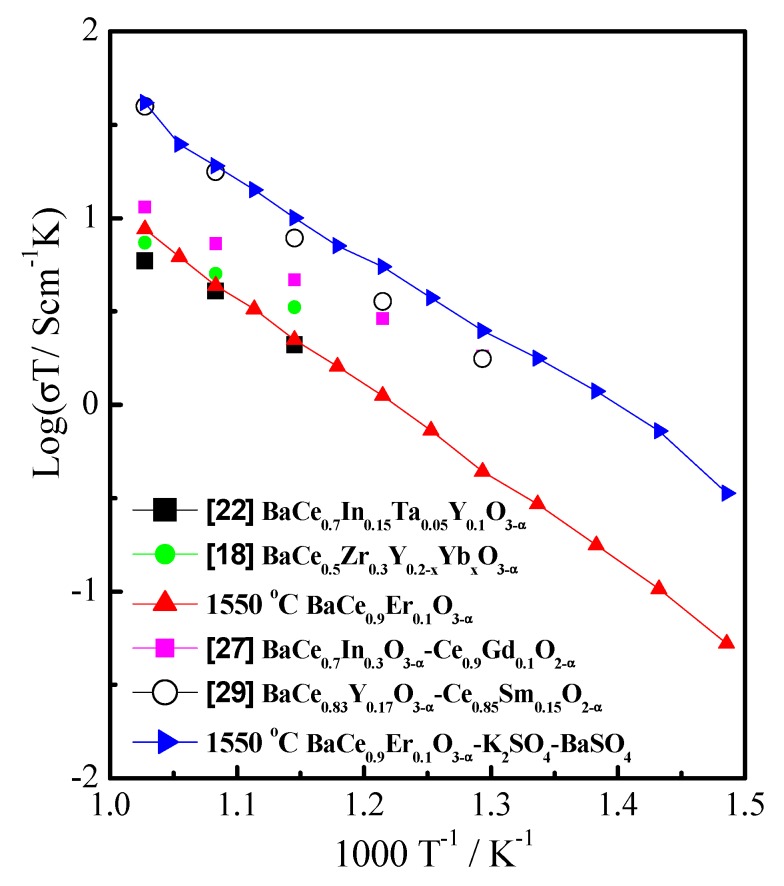
The conductivities of BaCe_0.9_Er_0.1_O_3−α_ (1550 °C) and BaCe_0.9_Er_0.1_O_3−α_–K_2_SO_4_–BaSO_4_ in nitrogen from 400 to 700 °C.

Figure 5 shows the conductivities of BaCe_0.9_Er_0.1_O_3−α_ (1550 °C) and BaCe_0.9_Er_0.1_O_3−α_–K_2_SO_4_–BaSO_4_ in nitrogen measured from 400 to 700 °C. It can be seen from Figure 5 that the BaCe_0.9_Er_0.1_O_3−α_–K_2_SO_4_–BaSO_4_ had a beneficial effect on conductivity. With the addition of sulphate, the conductivity was significantly improved. This is because the sulphate distributed at the grain boundary and formed a continuous phase, so both the main phase and the grain boundary phase could conduct ions. The highest conductivities of BaCe_0.9_Er_0.1_O_3−α_ (1550 °C) and BaCe_0.9_Er_0.1_O_3−α_–K_2_SO_4_–BaSO_4_ achieved were 9.4 × 10^−3^ and 1.8 × 10^−1^ S·cm^−1^ at 700 °C. Under the same conditions, the conductivity of BaCe_0.9_Er_0.1_O_3−α_–K_2_SO_4_–BaSO_4_ was higher than that of BaCe_0.7_In_0.3_O_3−δ_–Gd_0.1_Ce_0.9_O_2−δ_ [27] and comparable to values of BaCe_0.83_Y_0.17_O_3−δ_–Sm_0.15_Ce_0.85_O_2−δ_ [29]. This indicated that the sulphate was conducive to the conduction of ion defects through the interface region in the BaCe_0.9_Er_0.1_O_3−α_–K_2_SO_4_–BaSO_4_ composite electrolyte. The conductivity of BaCe_0.9_Er_0.1_O_3−α_ was equivalent to that of BaCe_0.7_In_0.15_Ta_0.05_Y_0.1_O_3−δ_ [22] and BaCe_0.5_Zr_0.3_Y_0.2−x_Yb_x_O_3−δ_ in wet H_2_ (~3% H_2_O) [18]. This may be related to its high density, as shown in Figure 3.

**Figure 6:**
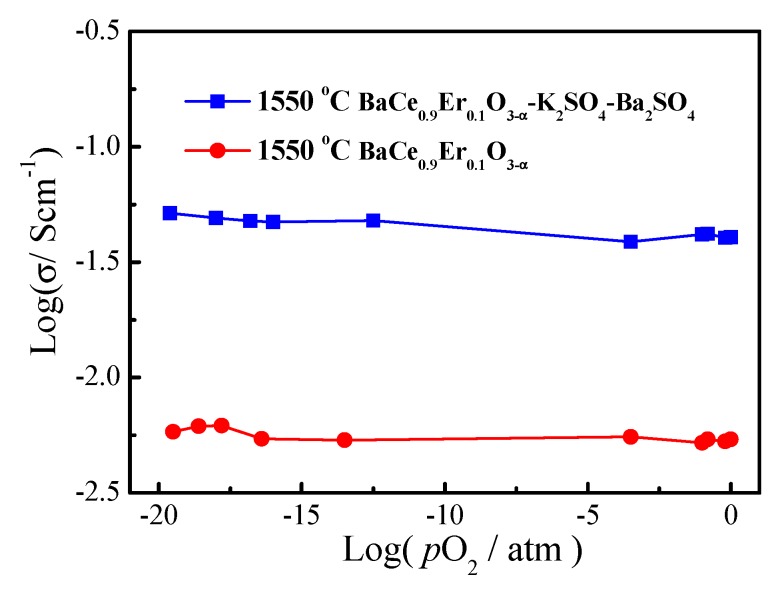
The logσ~log (*p*_O_2__) curves of BaCe_0.9_Er_0.1_O_3-α_ and BaCe_0.9_Er_0.1_O_3−α_–K_2_SO_4_–BaSO_4_ at 700 °C.

The conduction characteristics of BaCe_0.9_Er_0.1_O_3−α_ and BaCe_0.9_Er_0.1_O_3−α_–K_2_SO_4_–BaSO_4_ were tested by adjusting different proportions of gases. As seen in Figure 6, the conductivities of the samples in a reductive atmosphere are very close to those in an oxidizing atmosphere. The logσ~log (*p*_O_2__) curves of BaCe_0.9_Er_0.1_O_3−α_ and BaCe_0.9_Er_0.1_O_3−α_–K_2_SO_4_–BaSO_4_ were almost horizontal straight lines. When the temperature exceeded the melting point of sulphate salts, the mobility of various ions (Ba^2+^, Li^+^, K^+^, H^+^) was greatly enhanced, which led to a low activation energy for ion transport in the interface regions. The proton was the smallest cation, and the mobility of protons was greater than other ions (Li^+^, K^+^), resulting in an increased conductivity. Therefore, ion conduction appeared to become dominant [34].

**Figure 7:**
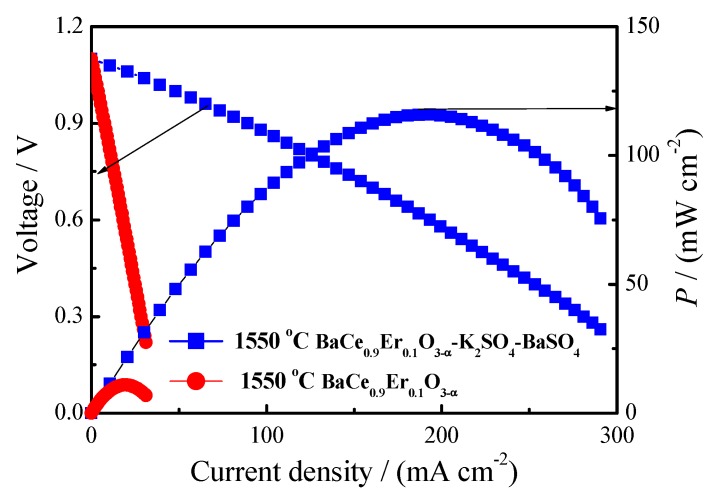
Hydrogen/oxygen fuel cells assembled with BaCe_0.9_Er_0.1_O_3-α_ and BaCe_0.9_Er_0.1_O_3−α_–K_2_SO_4_–BaSO_4_ as supporting electrolytes at 700 °C.

Hydrogen/oxygen fuel cells were assembled with BaCe_0.9_Er_0.1_O_3−α_ and BaCe_0.9_Er_0.1_O_3−α_–K_2_SO_4_–BaSO_4_ as supporting electrolytes and Pd–Ag as electrodes. The current–voltage characteristic curves are shown in Figure 7. The resistance directed from current–voltage characteristic curve of BaCe_0.9_Er_0.1_O_3−α_–K_2_SO_4_–BaSO_4_ (2.76 Ω) was lower than that of the value (5.54 Ω) from AC impedance at 700 °C, implying that the protonic conduction was dominant under the fuel cell condition [45]. The maximum power density of BaCe_0.9_Er_0.1_O_3−α_ was 10.9 mW·cm^−2^ at 700 °C. Because the fuel cell was supported by the electrolyte and the electrolyte was thicker (1.0 mm), the current and power density were relatively low. When the voltage was 0.6 V, the maximum output power density of BaCe_0.9_Er_0.1_O_3−α_–K_2_SO_4_–BaSO_4_ was 115.9 mW·cm^−2^ at 700 °C, which is ten times higher than that of BaCe_0.9_Er_0.1_O_3−α_. The results show that BaCe_0.9_Er_0.1_O_3−α_–K_2_SO_4_–BaSO_4_ is an excellent electrolyte material for medium-temperature fuel cells.

## 4. Conclusions

In this study, a BaCe_0.9_Er_0.1_O_3−α_–K_2_SO_4_–BaSO_4_ composite electrolyte was obtained by compounding it with a K_2_SO_4_–Li_2_SO_4_ solid solution. The XRD diffraction peaks of BaCe_0.9_Er_0.1_O_3−α_ (1550 °C) corresponded to the standard diagram of BaCeO_3_, which indicated that Er_2_O_3_ had entered the lattice of perovskite phase. SEM photos showed the BaCe_0.9_Er_0.1_O_3−α_ grains were wrapped in molten sulfate. The highest conductivities of BaCe_0.9_Er_0.1_O_3−α_ (1550 °C) and BaCe_0.9_Er_0.1_O_3−α_–K_2_SO_4_–BaSO_4_ were 9.4 × 10^−3^ and 1.8 × 10^−1^ S·cm^−1^ at 700 °C, respectively. The logσ~log (*p p*_O_2__) curves of BaCe_0.9_Er_0.1_O_3−α_ and BaCe_0.9_Er_0.1_O_3−α_–K_2_SO_4_–BaSO_4_ are almost horizontal straight lines, which indicated that ionic conductivity was dominant. The maximum output power density of BaCe_0.9_Er_0.1_O_3−α_–K_2_SO_4_–BaSO_4_ was 115.9 mW·cm^−2^ at 700 °C, which is ten times higher than that of BaCe_0.9_Er_0.1_O_3−α_.
